# Individual-and community-level factors associated with anemia among children aged 6–23 months in sub-Saharan Africa: evidence from 32 sub-Saharan African countries

**DOI:** 10.1186/s13690-022-00950-y

**Published:** 2022-08-06

**Authors:** Beminate Lemma Seifu, Getayeneh Antehunegn Tesema

**Affiliations:** 1 Department of Public Health, College of Medicine and Health Sciences, Samara University, Samara, Ethiopia; 2 Department of Epidemiology and Biostatistics, Institute of Public Health, College of Medicine and Health Sciences and Comprehensive Specialized Hospital, University of Gondar, Gondar, Ethiopia

**Keywords:** Anaemia, Children aged 6–23 months, SSA, Multilevel ordinal logistic regression analysis

## Abstract

**Background:**

Anemia among children aged 6–23 months is a major public health problem worldwide specifically in sub-Saharan Africa (SSA). Anemia during the childhood period causes significant short-and long-term health consequences. However, there is a paucity of evidence on Anemia among children aged 6–23 months in SSA. Therefore, this study examined the individual- and community-level factors associated with anemia among children aged 6–23 months in sub-Saharan Africa.

**Methods:**

A secondary data analysis was done based on the most recent Demographic and Health Survey (DHS) of 32 sub-Saharan African countries. A total weighted sample of 51,044 children aged 6–23 months was included for analysis. We have used a multilevel proportional odds model to identify predictors of severity levels of anemia. Variables with *p* < 0.2 in the bivariable analysis were considered for the multivariable analysis. In the multivariable multilevel proportional odds model, the Adjusted Odds Ratio (AOR) with the 95% Confidence Interval (CI) was reported to declare the statistical significance and strength of the association.

**Results:**

In this study, about 76.6% (95% CI: 76.2%, 76.9%) of children aged 6–23 months in sub-Saharan Africa were anemic. In the multivariable multilevel proportional odds model, being female, being aged 18–23 months, higher level of maternal education, being larger size at birth, belonging to a wealthier household, getting four ANC visits and above, advanced maternal age, and belonging to a community with high maternal education were significantly associated with lower odds of higher levels of anemia. On the other hand, being twin birth, being smaller size at birth, being of a higher order of birth, having fever in the last two weeks, and distance to a health facility were significantly associated with higher odds of higher levels of anemia.

**Conclusion:**

The study found that more than three-fourths of children aged 6–23 months in sub-Saharan Africa were anemic. This finding proved that the severity levels of anemia among children in sub-Saharan Africa remain a serious public health concern. Therefore, to curve this problem enhancing maternal education, promoting maternal health service utilization, and improving health care access is crucial. In addition, health care providers better give special emphasis to twin births, higher-order birth, and those belonging to poor households to reduce the incidence of anemia among children aged 6–23 months in SSA.

## Background

Globally, micronutrient deficiencies are the most prevalent nutritional problem among children [1, 2]. Anemia among children is the commonest micronutrient deficiency especially in sub-Saharan African countries [3]. According to the World Health Organization (WHO), anemia among children is defined as a hemoglobin value less than 11 g/dl [4]. Anemia affects all age groups but under two years children are the most vulnerable segment of the community [5, 6]. In the first two years of life, the child has rapid mental development and physical growth which demands the highest nutritional supply [7]. As of 2019, an estimated 269 million children suffered from anemia with nearly two-thirds in Asia and Africa [8, 9]. The highest prevalence of anemia among children was observed in sub-Saharan Africa (46%—66%) [3, 10, 11].

Anemia during childhood has short-and long-term consequences on physical growth, mental development, and productivity [12–14]. It causes mental retardation [15], poor physical performance [16], and poor motor development and control [17]. In the long term, it leads to reduced academic achievement [18–20]. The causes of anemia can be nutritional, medical, or genetic [21]. Nevertheless, nutritional deficiency is the commonest cause of anemia specifically iron deficiency [22]. Apart from ID, other micronutrient deficiencies (folic acid, zinc, and vitamin B12) [23], parasitic infections (hookworm, schistosomiasis, ascariasis, and malaria) [24], and blood disorders (sickle cell anemia and thalassemia) [25] can cause anemia.

Previous studies revealed that residence [26], mothers age [27], mothers educational status [28], child age [29], child sex [30], child twin status [31], number of Antenatal Care (ANC) visits [32, 33], place of delivery [34], birth weight [35], media exposure [11], distance to health facility [36], household wealth status [37, 38], birth order [39], maternal anemia [40], infectious diseases (malaria, tuberculosis, and hookworm) [41], and child nutritional status (stunting, wasting and underweight) [42] were significant factors associated with anemia.

Studies have proven that anemia among children aged 6–23 months is a serious public health problem in SSA. However, there is a paucity of evidence on the prevalence, severity level, and predictors of anemia among children aged 6–23 months in SSA. In addition, even though anemia has ordinal nature (not anemic, mild, moderate, or severe), previous studies treat anemia as a binary outcome (anemic vs non-anemic). Therefore, treating anemia as binary could result in loss of information and is not informative for decision-makers as well as public health programmers. Therefore, this study examined the individual-and community-level factors associated with anemia among children aged 6–23 months in SSA using a multilevel ordinal logistic regression model.

## Methods

### Data source and sampling procedure

This study was based on the most recent Demographic and Health Survey (DHS) of 32 sub-Saharan African countries. DHS is conducted every five years to generate updated health and health-related indicators. A two-stage stratified sampling technique was employed to select the study subjects. In the first stage, Enumeration Areas (EAs) were randomly selected while in the second stage households were selected.

To assess the anemia status of children, hemoglobin testing was carried out among children in the selected households using HemoCue rapid testing methodology. For the test, a drop of capillary blood was taken from a child's fingertip or heel and was drawn into the micro cuvette which was then analyzed using the photometer that displays the hemoglobin concentration. There are different datasets in DHS and for this study, we used the Kids Record (KR) file. We extracted the data from the KR dataset based on literature and then appended using the STATA command “append using”. The final sample size for this study was 51,044 children aged 6–23 months.

### Study variables and measurements

#### Dependent variable

Our outcome variable of interest was severity levels of anemia among children aged 6–23 months, which we grouped into four ordinal categories; mildly anemic (hemoglobin level 10.0–10.9 g/dl, moderately anemic (hemoglobin level 7.0–9.9 g/dl), severely anemic (hemoglobin level < 7.0 g/dl), and not anemic (hemoglobin ≥ 11.0 g/dl). It was assessed based on the hemoglobin concentration in blood adjusted to the altitude. At elevations above 1000 m, hemoglobin concentrations increase as an adaptive response to the lower partial pressure of oxygen and reduced oxygen saturation of the blood. The compensatory increase in red cell production ensures that sufficient oxygen is supplied to tissues.

$${\mathrm{Hb}=-0.32\times\left(\mathrm{altitude}\;\mathrm{in}\;\mathrm{meters}\times.0033\right)+0.22\times\left(\mathrm{altitude}\;\mathrm{in}\;\mathrm{meters}\times.0033\right)}^2$$

#### Independent variables

The independent variables were considered from two sources; individual-and community-level variables. Child age, child sex, household wealth status, maternal education, maternal age, sex of household head, child twin status, child nutrition status, birth order, media exposure, number of ANC visits, place of delivery, fever in the last two weeks, husband education, had diarrhea in the last two weeks, birth size, and preceding birth interval were individual-level variables. Residence, sub-Saharan African region, distance to the health facility, community poverty, and community maternal education were community-level variables.

Media exposure was calculated by aggregating three variables such as watching television, listening to the radio, and reading newspapers. Then categorized as having media exposure if a mother has been exposed to at least one of the three and not if she had no exposure to any of the media sources. These two community-level variables (community maternal education and community poverty) were generated by aggregating maternal education and household wealth status at the cluster/enumeration area levels. Then categorized as higher community maternal education and poverty based on the national median value of the proportion of maternal education and poverty since they were not normally distributed.

### Data management and analysis

STATA version 17 statistical software was used for the data management and analysis. anemia was polychotomous and has ordinal nature. Therefore, an ordinal logistic regression model was considered to analyze factors associated with severity levels of anemia (no anemia, mild, moderate, and severe anemia). There are different ordinal logistic regression models such as Proportional Odds Model (POM), Partial Proportional Odds Model with Restrictions (PPOMR), Partial Proportional Odds Model without Restrictions (PPOMWR), Continuous Ratio Model (CRM), and Stereotype Model (SM). In epidemiological studies, Proportional Odds Model (Cumulative Logit Model) is the most commonly used ordinal logistic regression model. The fundamental assumption in an ordinal logistic regression model is the Proportional Odds (PO) assumption. If the data satisfies the PO assumption, the proportional odds model can be used, if not the partial proportional model should be used. To choose the appropriate ordinal model for the data, we have checked the PO assumptions, which state that the effects of all independent variables are constant across categories of the outcome variable. The Brant test revealed that the PO assumption was fulfilled (*p* > 0.05). Besides, the DHS data has a hierarchical nature. Therefore, children and mothers were nested within a cluster, and we assume that study subjects in the same cluster may share similar characteristics to participants in another cluster. This implies the need to take into account the heterogeneity between clusters by using an advanced model. Therefore, a multilevel proportional odds model was performed.

As a result, because the Brant test was met, the multilevel proportional odds model gave a single Odds Ratio (OR) for an explanatory variable (severe vs moderate/mild/non-anemia, severe/moderate vs mild/non-anemia, and severe/moderate/mild vs non-anemic. The Brant test stated that the effects of all the independent variables are constant across categories of severity levels of anemia (global test; *p* > 0.05). In addition to the global test, we assessed each variable in the model to identify the variables for which the PO assumption was fulfilled and all the variables had a *p*-value > 0.05. Likelihood Ratio (LR) test, Variance Partition Coefficient (VPC), and Median Odds Ratio (MOR) were computed to measure the variation of anemia across clusters. The VPC quantifies the degree of heterogeneity of anemia between clusters (the proportion of the total observed variation in anemia that is attributable to cluster variations) [43].

$$\mathrm{VPC}={6}^{2}/\left({6}^{2}+{\pi }^{2}/3\right)$$

Where: the standard logit distribution has a variance of ${\uppi }^2/3$, ${{\sigma }_{\mu }}^{2}$ indicates the cluster variance.

The MOR quantifies the variation or heterogeneity in anemia between clusters in terms of the odds ratio scale and is defined as the median value of the odds ratio between the cluster with a high likelihood of anemia and the cluster at lower risk when randomly picking out individuals from two clusters (EAs) [44].

$$\mathrm{MOR}\hspace{0.17em}=\hspace{0.17em}{exp}^{\surd ( 2*\partial 2*0.6745 )},\mathrm{ MOR}= {exp}^{0.95*\partial }$$

Four models were constructed for the multilevel logistic regression analysis. The first model was a null model without explanatory variables to determine the extent of cluster variation in childhood anemia. The second model was adjusted with individual-level variables; the third model was adjusted for community-level variables while the fourth was fitted with both individual and community-level variables simultaneously. Model comparison was made based on deviance (-2Log-Likelihood Ratio (LLR)) since the models were nested models, and a model with the lowest deviance was the best-fitted model for the data.

In the null model, about 7.4% of the total variation in anemia was due to unobserved community-level factors (VPC = 7.4%). This result was not suggestive of using a multilevel ordinal model rather than a single-level model. Then, we have further examined whether the multilevel ordinal logistic regression model was significant over the single-level ordinal logistic regression model using the LR test as VPC is descriptive and we don’t rely on its value as it is not directly estimated, unlike continuous outcome variables. The LR test was statistically significant (*p* < 0.05), indicating that the multilevel ordinal logistic regression model was best fitted to the single-level ordinal logistic regression analysis. In addition, MOR revealed that if we randomly select two children aged 6–23 months from different clusters and transfer children aged 6–23 months from the cluster with a lower likelihood of higher levels of anemia to a cluster with a higher likelihood of higher levels of anemia, could have 1.32 times higher levels of anemia. Therefore, the LR-test and MOR are suggestive of using a multilevel ordinal logistic regression model. Four models were fitted and the final model was chosen since it has the lowest deviance value.

Variables with a *p*-value ≤ 0.2 in the bi-variable multilevel proportional odds model were considered for the multivariable multilevel proportional odds model. In the multivariable multilevel proportional odds model, the Adjusted Odds Ratio (AOR) with 95% Confidence Interval (CI) was reported to declare the strength of association, and the statistical significance for the final model was set at *p* < 0.05.

### Ethical consideration

This study was based on publicly accessible survey data from the MEASURE DHS program, and no need for ethical approval and participant consent. We have granted permission from http:/www.dhsprogram.com to download and use the data for this study. In the datasets, there are no names of persons or household addresses recorded.

## Results

### Individual-level characteristics of study participants

A total of 51,044 children aged 6–23 months were included. Of them, about 25,664 (50.3%) were males. More than half (52.5%) of mothers were aged 20–29 years and about 36.6% of mothers didn’t attend formal education. About 27.3% and 25.4% of the children had symptoms of fever and diarrhea within two weeks, respectively. About 53.5% of the mothers had four ANC visits and above during pregnancy, and the vast majority (70.5%) of the children were born at the health facility (Table 1).

**Table 1:** Individual-level characteristics of the study participants in sub-Saharan Africa (*n* = 51,044)

Variable	Weighted frequency	Percentage (%)
**Child sex**		
Male	25,664	50.3
Female	25,380	49.7
**Child age (in months)**		
6–11	17,357	34.0
12–17	18,212	35.7
18–23	15,475	30.3
**Child twin status**		
Single	49,382	96.7
Multiple	1662	3.3
**Maternal age**		
15–19	4808	9.4
20–29	26,412	51.7
30–29	16,589	32.5
40–49	3235	6.3
**Maternal education status**		
No	18,657	36.6
Primary	18,195	35.6
Secondary	12,828	25.1
Higher	1364	2.7
**Child size at birth**		
Average	24,439	47.9
Smaller than average	17,782	34.8
Larger than average	8823	17.3
**Media exposure**		
No	17,270	33.8
Yes	33,774	66.2
**Birth order**		
1^st^	11,359	22.2
2^nd^ or 3^rd^	18,159	35.6
4^th^ or 5^th^	11,493	22.5
Above 6^th^	10,043	19.7
**Stunting status (*n* = 49,311)**		
Normal	33,515	68.0
Moderate	9668	19.6
Severe	6128	12.4
**Wasting status (*n* = 47,916)**		
Normal	43,060	89.9
Moderate	3467	7.2
Severe	1388	2.9
**Underweight status (*n* = 48,098)**		
Normal	39,255	81.6
Moderate	6307	13.1
Severe	2536	5.3
**Household wealth status**		
Poorest	11,441	22.4
Poorer	11,069	21.7
Middle	10,498	20.6
Richer	9851	19.3
Richest	8185	16.0
**Had a fever in the last two weeks**		
No	37,094	72.7
Yes	13,950	27.3
**Had diarrhea in the last two weeks**		
No	38,099	79.7
Yes	12,945	25.4
**Sex of household head**		
Male	40,696	79.7
Female	10,348	20.3
**Husband's educational status (*n* = 45,728)**		
No	15,878	34.7
Primary	14,035	30.7
Secondary	13,042	28.5
Higher	2773	6.1
**Preceding birth intervals in months (*n* = 39,385)**		
< 24	6260	15.8
24–59	26,920	68.0
≥ 60	6404	16.2
**Number of ANC visit**		
No	6804	13.3
1–3	16,944	33.2
≥ 4	27,296	53.5
**Place of delivery**		
Home	15,048	29.5
Health facility	35,996	70.5

### Community-level characteristics of the study participants

The majority of the children were from West Africa (39.9%) followed by East Africa (38.6%). More than two-thirds (69.0%) of respondents were living in rural areas. Regarding community maternal education and poverty, about 27,580 (54.0%) and 3,639 (7.1%) of the respondents were from the community with high maternal education and high poverty, respectively (Table 2).

**Table 2:** Community-level characteristics of the respondents in sub-Saharan Africa

Variable	Weighted frequency (*n* =)	Percentage (%)
**Sub-Saharan Africa**		
East Africa	19,690	38.6
West Africa	20,388	39.9
Southern Africa	2012	3.9
Central Africa	8954	17.5
**Residence**		
Urban	15,809	31.0
Rural	35,235	69.0
**Distance of health facility**		
Not a big problem	30,557	59.9
Big problem	20,487	40.1
**Community poverty**		
Low	47,405	92.9
High	3639	7.1
**Community maternal education**		
Low	23,464	46.0
High	27,580	54.0

### Prevalence of anemia among children aged 6–23 months in SSA

**Fig. 1:**
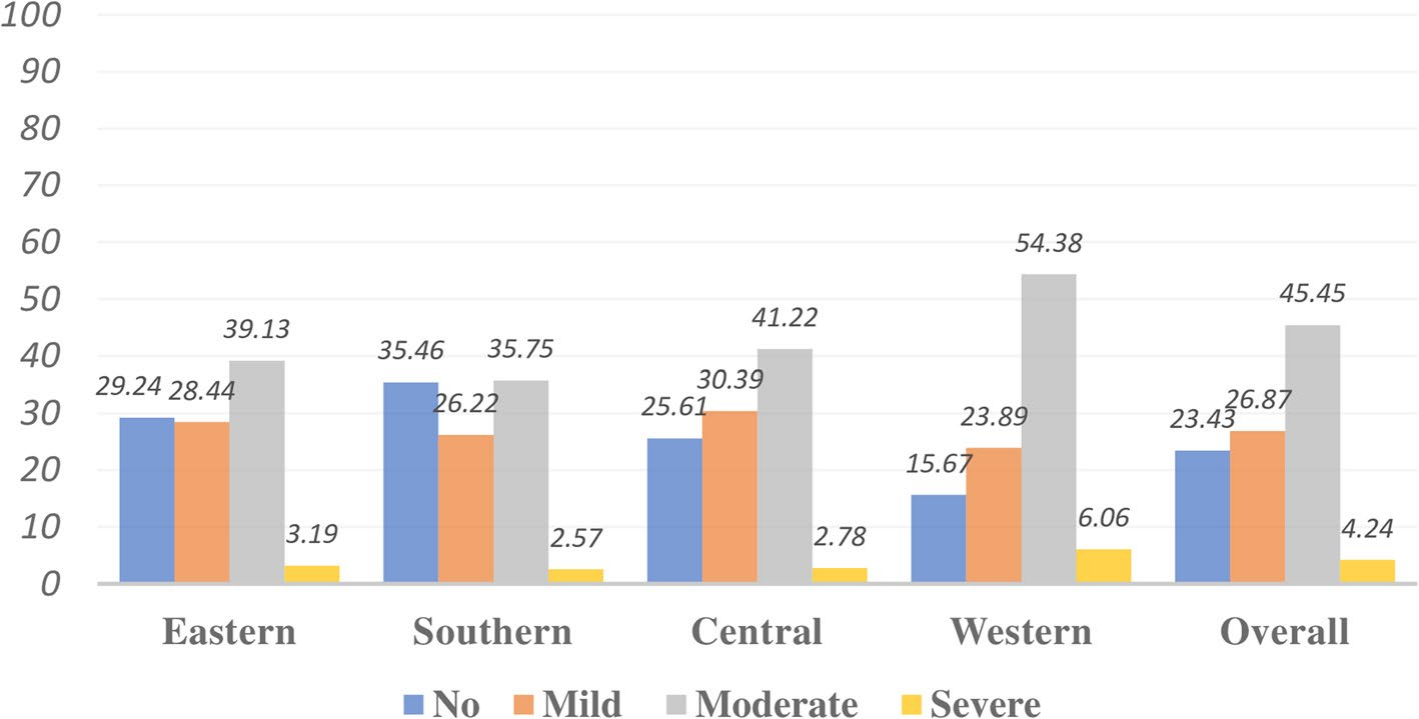
The prevalence of severity levels of anemia among children aged 6–23 months in sub-Saharan African regions

The prevalence of anemia among children aged 6–23 months in SSA was 76.6% (95% CI: 76.2%, 76.9%). The prevalence varied from 64.5% in Southern Africa to 84.3% in West Africa. Based on severity levels of anemia, the prevalence of mild, moderate and severe anemia among children aged 6–23 months in SSA were 26.9% (95% CI: 26.5%, 27.3%), 45.5% (95% CI: 45.0%, 45.9%) and 4.2% (95% CI: 4.1%, 4.4%), respectively (Fig. 1).

### Multivariable multilevel proportional odds model analysis results

#### Individual and community-level factors associated with anemia

Sex of child, child age, maternal education, larger size at birth, household wealth status, number of ANC visits, maternal age, being in southern Africa, and higher community maternal education was significantly associated with lower risk of higher levels of anemia. In contrast, twin status, smaller size at birth, birth order, health facility delivery, and being born in Central and West Africa were significantly associated with higher risks of higher levels of anemia.

The odds of having higher levels of anemia among female children decreased by 22% (AOR = 0.78, 95% CI: 0.76, 0.81) compared to male children. The odds of having higher levels of anemia were decreased by 27% (AOR = 0.73, 95% CI: 0.70, 0.76) for children aged 18–23 months compared to children aged 6–11 months. The odds of having higher levels of anemia among children of mothers who attained a primary, secondary and higher level of education were decreased by 20% (AOR = 0.80, 95% CI: 0.77, 0.84), 30% (AOR = 0.70, 95% CI: 0.66, 0.74) and 51% (AOR = 0.49, 95% CI: 0.43, 0.55) compared to children of mothers who didn’t have formal education, respectively.

Children who were smaller size at birth were 1.16 times (AOR = 1.16, 95% CI: 1.11, 1.21) times higher odds of having higher levels of anemia compared to those of average size at birth. On the other hand, the odds of having higher levels of anemia among children with a larger size at birth were decreased by 9% (AOR = 0.91, 95% CI: 0.88, 0.95) than those average size at birth. Being sixth birth order or above had 1.12 times (AOR = 1.12, 95% CI: 1.04, 1.20) higher odds of higher levels of anemia compared to first order birth. The odds of having higher levels of anemia among children belonged to the middle, richer, and richest household wealth were decreased by 9% (AOR = 0.89, 95% CI: 0.87, 0.96), 9% (AOR = 0.91, 95% CI: 0.86, 0.96) and 22% (AOR = 0.78, 95% CI: 0.73, 0.84) compared to children belonging to the poorest household wealth, respectively.

Children who had a fever in the last two weeks had 1.40 times (AOR = 1.40, 95% CI: 1.35, 1.45) higher odds of higher levels of anemia compared to those who didn’t have a fever. The odds of having higher levels of anemia among children born to mothers aged 20–29, 30–39 and 40–49 years were decreased by 14% (AOR = 0.86, 95% CI: 0.80, 0.91), 27% (AOR = 0.73, 95% CI: 0.68, 0.79) and 36% (AOR = 0.64, 95% CI: 0.58, 0.71) compared to children born to mothers aged 15–19 years, respectively. The odds of having higher levels of anemia among children born to mothers who had four and above ANC visits were decreased by 9% (AOR = 0.91, 95% CI: 0.86, 0.96) compared to children born to children whose mothers didn’t have ANC visits during pregnancy.

Children born to mothers who reported distance to a health facility as a big problem had 1.06 times (AOR = 1.06, 95% CI: 1.03, 1.10) higher odds of higher levels of anemia compared to their counterparts. Children in Central Africa and West Africa had 1.17 times (AOR = 1.17, 95% CI: 1.12, 1.23) and 2.09 times (AOR = 2.09, 95% CI: 2.01, 2.18) higher odds of higher levels of anemia than those in East Africa whereas children in Southern Africa had decreased odds of higher levels of anemia (AOR = 0.89, 95% CI: 0.82, 0.97) than children in East Africa. The odds of having higher levels of anemia among children in the community with higher maternal education were decreased by 8% (AOR = 0.92, 95% CI: 0.86, 0.99) compared to children in the community with low maternal education (Table 3).

**Table 3:** Multilevel ordinal logistic regression analysis of individual and community level variables associated with severity levels of anemia among children aged 6–23 months in sub-Saharan Africa

Variables	Null model	Model I	Model II	Model III
**Sex of child**				
Male		1		1
Female		0.78 (0.76, 0.81)		0.78 (0.76, 0.81)^**^
**Child age**				
6–11		1		1
12–17		0.96 (0.93, 1.01)		0.97 (0.93, 1.01)
18–23		0.73 (0.70, 0.76)		0.73 (0.70, 0.76)^**^
**Child twin status**				
Single		1		1
Twin		1.63 (1.48, 1.88)		1.53 (1.39, 1.69)^*^
**Maternal educational status**				
No		1		1
Primary		0.59 (0.57, 0.61)		0.80 (0.77, 0.84)^*^
Secondary		0.53 (0.51, 0.56)		0.70 (0.66, 0.74)^**^
Higher		0.38 (0.34, 0.43)		0.49 (0.43, 0.55)^*^
**Chid size at birth**				
Average		1		1
Larger than average		0.94 (0.91, 0.98)		0.91 (0.88, 0.95)^*^
Smaller than average		1.16 (1.11, 1.21)		1.16 (1.11, 1.21)^**^
**Media exposure**				
No		1		1
Yes		1.06 (1.03, 1.11)		0.98 (0.94, 1.02)
**Birth order**				
1^st^		1		1
2^nd^ – 3^rd^		1.00 (0.95, 1.05)		1.01 (0.96, 1.07)
4^th^ -5^th^		1.04 (0.97, 1.10)		1.05 (0.99, 1.12)
6^th^ and above		1.08 (1.00, 1.16)		1.12 (1.04, 1.20)^*^
**Household wealth status**				
Poorest		1		1
Poorer		1.01 (0.96, 1.06)		0.98 (0.93, 1.03)
Middle		0.97 (0.92, 1.02)		0.91 (0.87, 0.96)^*^
Richer		0.97 (0.92, 1.02)		0.91 (0.86, 0.96)^*^
Richest		0.86 (0.81, 0.92)		0.78 (0.73, 0.84)^*^
**Had a fever for the last two weeks**				
No		1		1
Yes		1.39 (1.34, 1.44)		1.40 (1.35, 1.45)^**^
**Number of ANC visits**				
No		1		1
1–3		1.07 (1.01, 1.13)		1.06 (0.99, 1.12)
≥ 4		0.93 (0.88, 0.99)		0.91 (0.86, 0.96)^*^
**Place of delivery**				
Home		1		1
Health facility		0.98 (0.94, 1.02)		1.04 (0.99, 1.08)
**Maternal age**				
15–19		1		1
20–29		0.87 (0.82, 0.93)		0.86 (0.80, 0.91)^*^
30–39		0.75 (0.69, 0.81)		0.73 (0.68, 0.79)^*^
40–49		0.66 (0.59, 0.73)		0.64 (0.58, 0.71)^*^
**Residence**				
Urban			1	1
Rural			1.26 (1.21, 1.31)	0.99 (0.95, 1.04)
**Sub-Saharan African region**				
East			1	1
Southern			0.84 (0.77, 0.91)	0.89 (0.82, 0.97)^*^
Central			1.21 (1.16, 1.27)	1.17 (1.12, 1.23)^*^
West			2.29 (2.20, 2.37)	2.09 (2.01, 2.18)^*^
**Distance to the health facility**				
Not a big problem			1	1
Big problem			1.13 (1.09, 1.17)	1.06 (1.03, 1.10)^*^
**Community maternal education**				
Low			1	1
High			0.80 (0.75, 0.86)	0.92 (0.86, 0.99)^*^
**Community poverty**				
Low			1	1
High			0.99 (0.95, 1.03)	0.97 (0.93, 1.01)
/cut1	0.31 (0.30, 0.32)	-1.92 (-2.01, -1.84)	-0.68 (-0.73, -0.64)	-1.53 (-1.63, -1.42)
/cut2	0.01 (0.009, 0.03)	-0.68 (-0.77, -0.59)	0.55 (0.51, 0.60)	-0.26 (-0.37, -0.15)
/cut3	3..14 (3.10, 3.19)	2.54 (2.44, 2.63)	3.76 (3.69, 3.82)	3.00 (2.89, 3.12)
**Random effect analysis result**				
Community-level variance	0.25	0.24	0.22	0.20
LR test	Prob > = chibar2 < 0.01			
VPC (%)	7.4	6.8	6.3	5.7
MOR	1.32	1.30	1.24	1.19
LLR	-60,800.71	-59,483	-59,651.73	-58,795.65
Deviance	121,601.42	118,966	119,303.46	117,591.3
AIC	121,609.4	119,022	119,325.5	117,591.3
BIC	121,644.8	119,269.7	119,422.8	117,970.9

*AIC* Akaike Information Criteria, *BIC* Bayesian Information Criteria, *LLR* Log-likelihood Ratio, *LR* Likelihood Ratio, *MOR* Median Odds Ratio, *VPC* Variance Partition Coefficient

## Discussion

The prevalence of anemia among children aged 6–23 months in SSA was 76.6% (95% CI: 76.2%, 76.9%) which varied across regions, indicating anemia is a serious public health problem in SSA [45]. This was higher than in previous studies [46–50], this might be due to the long-standing undernutrition among children due to long-standing poverty in SSA [51]. Moreover, poor socioeconomic conditions in African countries make children more susceptible to infectious diseases, which in turn compromises the absorption, and utilization of nutrients like vitamin B9, B12, and iron [52, 53].

Child sex, child age, maternal educational status, child size at birth, household wealth status, number of ANC visits, maternal age, having fever in the last two weeks, sub-Saharan African region, community maternal education, child twin status, birth order and distance to health facility were significantly associated with the severity levels of anemia. In line with this study [10, 54, 55], previous studies revealed that female children were at lower odds of having higher levels of anemia compared to male children. The possible justification might be due to too early initiation of complementary feeding for male children and the community’s belief to give better care to male children than females, this could expose them to different infectious diseases and malabsorption problems [56, 57]. In addition, male children in the first two years of life are at a state of rapid growth which increases their micronutrient demands including vitamin B9, B12, and iron compared to female children [58, 59].

Twin children were at higher odds of having higher levels of anemia compared to singletons. This is supported by a study reported in India [60], this might be due to twin births being prone to prematurity, low birth weight, and undernutrition than singletons, in turn, they are at higher risk of anemia [61, 62]. Children aged 18–23 months had lower odds of higher levels of anemia compared to children aged 6–11 months. This is in line with previous studies reported in developing countries [54, 63], this is because prenatal iron store depletion is pronounced at six months of birth and made them at higher risk of anemia [64].

As the level of maternal education increased, the odds of higher levels of anemia decreased. It is consistent with studies reported in Korea [63], Indonesia [65], and Mexico [66]. This might be because children of mothers who had formal education are more likely to have healthy child-feeding practices [67, 68]. In addition, a mother with a higher level of education has better nutrition and utilizes health services [69, 70]. Many studies have reported maternal anemia as the commonest risk factor for anemia among children [40, 71]. Being small size at birth was significantly associated with increased odds of higher levels of anemia while large size at birth was significantly associated with lower odds of higher levels of anemia. These were in agreement with studies reported in India [72] and Pakistan [73], this could be due to small size at birth being associated with maternal anemia [74], which in turn children born to anemic mothers may not have enough iron storage [40, 75].

Children from households with middle, richer, and richest wealth quantile had decreased odds of having higher levels of anemia compared to those from a household with the poorest wealth quintile. This is consistent with study findings in SSA [39], Sri Lanka [76], and Bangladesh [77], this could be because children in the wealthiest household are capable of providing a balanced diet rich in macro and micronutrients including minerals and vitamins to their child [78, 79]. In addition, children from rich households have more chance of health care access for common illness which causes childhood anemia [80]. The odds of having higher levels of anemia among children born to mothers who had four and above ANC visits during pregnancy were lower than those born to mothers who didn’t have ANC visits. This might be due to pregnant mothers may have the benefit of iron-folate supplementation as well as prompt diagnosis and treatment of diseases such as malaria, visceral leishmaniasis, and hookworm which are identified as the leading causes of anemia, which in turn reduces the risk of child anemia [81, 82].

Children of mothers aged 20–29, 30–39, and 40–49 years had lower odds of higher levels of anemia compared to children of a mother aged 15–19 years. This was in agreement with study findings in Haiti [29], Brazil [83], and India [84], this might be due to mothers aged 20 years and above are physiologically mature and are at lower risk of having low birth weight babies compared to young women [85, 86]. Higher birth order was significantly associated with increased odds of having higher levels of anemia compared to first-order birth. This was in concordance with study findings in SSA [39], India [87, 88], this is because higher birth order is related to extensive maternal nutrition depletion including iron, folate, and vitamin B12 and this could increase the risk of childhood anemia [89, 90]. In addition, a large number of children are associated with increased socio-economic and health problems due to competition for food, infections, and cross contaminations.

Children who had a fever in the last two weeks had higher odds of higher levels of anemia compared to their counterparts. This is consistent with different studies reported elsewhere [11, 91, 92], this is could be due to fever is commonly manifested due to malaria and other common infectious diseases in a situation where both anemia and fever coexist [93, 94]. In agreement with previous studies [40, 95], the odds of having higher levels of anemia among children of mothers perceiving distance to a health facility as a big problem were higher than their counterparts. This might be due to the big distance to the nearby health facility being linked to poor health service utilization for basic services. To mention a few, malaria and hookworm infections are common in children aged 6–23 months in almost all African countries, and if they are not timely and effectively treated, they could result in a severe form of anemia.

## Strengths and limitations of the study

Our study findings should be interpreted within the context of the following limitation. We are unable to draw cause-effect relationships because of the cross-sectional nature of DHS data. In addition, in this study, only children surviving during the data collection were included and deaths that could have resulted from complications due to anemia were missed (survivor bias). Moreover, we were not considered important predictors such as malaria infection, visceral leishmaniasis, hookworm, and congenital infections, because these variables were not available in DHS. Despite the abovementioned limitations, this study has the following strengths. This study was based on a pooled nationally representative DHS survey of the 32 sub-Saharan African countries. In addition, the data was weighted and a multilevel ordinal logistic regression analysis was done to get a reliable estimate and standard error. Besides, this study was based on a large sample size that had adequate power to detect the true effect of the independent variables.

## Conclusion

Anemia among children aged 6–23 months in sub-Saharan Africa was a serious public health problem. Both individual and community level variables were found significant predictors of severity levels of anemia among children aged 6–23 months. Child age, child sex, size at birth, maternal education, maternal age, fever in the last two weeks, birth order, twin status, and household wealth status were among individual-level predictors of severity levels of anemia among children aged 6–23 months. Among community-level variables, sub-Saharan African regions, distance to health facilities, and community maternal education were found significant predictors of severity levels of anemia. Improving maternal education, providing treatment for febrile illness, and strengthening the economic status of the family are recommended to reduce anemia among children aged 6–23 months. In addition, to cater anemia fortification or supplementation with iron and micronutrients could contribute for the better reduction in the risk of anemia and improvements in hemoglobin levels among children.

## Data Availability

Data is available online and you can access it from www.measuredhs.com.

## Abbreviations

AIC: Akaike Information Criteria
AOR: Adjusted Odds Ratio
BIC: Bayesian Information Criteria
CI: Confidence Interval
DHS: Demographic health survey
EAs: Enumeration areas
ID: Iron Deficiency
HAZ: Z-score for Height-for-Age
IUGR: Intra uterine growth restriction
LLR: Log likelihood ratio
LR: Likelihood ratio
POM: Proportional Odds Model
PPOM: Partial Proportional Odds Model
SDG: Sustainable Development Goal
SSA: Sub-Saharan Africa;
WAZ: Z-score for Weight-for-Age
WHO: World Health Organizations
WHZ: Z-score for Weight-for-Height
